# Complete Tri-Axis Magnetometer Calibration with a Gyro Auxiliary

**DOI:** 10.3390/s17061223

**Published:** 2017-05-26

**Authors:** Deng Yang, Zheng You, Bin Li, Wenrui Duan, Binwen Yuan

**Affiliations:** 1 Department of Precision Instrument, Tsinghua University, Beijing 100084, China; yangd14@mails.tsinghua.edu.cn (D.Y.); lbin@mail.tsinghua.edu.cn (B.L.); duanwr@mail.tsinghua.edu.cn (W.D.); yuanbw15@mails.tsinghua.edu.cn (B.Y.); 2 State Key Laboratory of Precision Measurement Technology and Instruments, Tsinghua University, Beijing 100084, China

**Keywords:** tri-axis magnetometer, calibration, gyro auxiliary, interpolation, linear operation

## Abstract

Magnetometers combined with inertial sensors are widely used for orientation estimation, and calibrations are necessary to achieve high accuracy. This paper presents a complete tri-axis magnetometer calibration algorithm with a gyro auxiliary. The magnetic distortions and sensor errors, including the misalignment error between the magnetometer and assembled platform, are compensated after calibration. With the gyro auxiliary, the magnetometer linear interpolation outputs are calculated, and the error parameters are evaluated under linear operations of magnetometer interpolation outputs. The simulation and experiment are performed to illustrate the efficiency of the algorithm. After calibration, the heading errors calculated by magnetometers are reduced to 0.5° (1σ). This calibration algorithm can also be applied to tri-axis accelerometers whose error model is similar to tri-axis magnetometers.

## 1. Introduction

Strap-down inertial measurement units (SIMU) which consist of gyroscopes and accelerometers, have been applied to orientation estimation for a long time [1]. Nowadays, magnetometers rigidly mounted to SIMU, also known as MARG (Magnetic, Angular Rate, and Gravity), are popular for achieving better performance. However, the magnetometer outputs suffer from magnetic field distortions and sensor errors, including the hard iron effect, soft iron effect, zero bias error, scale factor error, non-orthogonal error, misalignment error, and noise [2]. Magnetometer calibrations are always advisable to be done prior to application.

The calibration methods which generally rely on non-magnetic turntables [3,4,5] and Helmholtz coils [6,7,8,9], are proposed first. The magnetometer error parameters can be precisely calibrated. However, the cumbersome processes and calibration platforms bring about the issues of inconvenience and expense. Many stand-alone magnetometer calibration algorithms based on an ellipse-fitting method have been put forward [10,11,12,13,14,15]. These algorithms are low-cost and easy to use. The aim of these ellipse-fitting based algorithms is to compensate the magnetometer output data from lying on an ellipsoid to a sphere. However, the misalignment error between magnetometers and assembled platforms, which causes a rotation of the sphere, is unable to be calibrated. For complete tri-axis magnetometer calibration, a series of calibration algorithms in combination with accelerometers [16,17,18,19,20,21] are proposed. First, the ellipsoid-fitting algorithms are performed to calibrate errors, except the misalignment error. Then, optimization problems based on the inner products of the tri-axis magnetometer outputs and accelerometer outputs are established and solved for misalignment error estimation. The author further applies gyroscopes with magnetometers and accelerometers to run an EKF (Extended Kalman Filter) for a maximum likelihood calibration process [22,23]. The nonlinear and iterative operations as well as the reliance on initial values create the drawback of heavy computation burdens for these algorithms. Tri-axis magnetometers and gyroscopes are also simultaneously applied in [24,25] to establish an EKF and an adaptive identification algorithm for magnetometer calibration without the use of an accelerometer. The error models of a tri-axis magnetometer are simplified. Only parts of the errors are calibrated, which degrade the calibration performance.

In this work, a tri-axis gyroscope is applied as an auxiliary for complete tri-axis magnetometer calibration. Firstly, the sensor set of the tri-axis magnetometer and the tri-axis gyroscope is mounted into a non-magnetic cuboid frame. Then, the frame is put on a table and rotated for at least one cycle for each side of the frame, respectively. Finally, the complete tri-axis magnetometer calibration is achieved through linear algebra operations of the magnetometer and gyroscope output data. After calibration, the heading errors calculated by magnetometers are reduced to 0.5° (1σ). This algorithm requires a cuboid frame to mount the sensor set, which is easy to obtain and inexpensive. The proposed method, which employs the linear algebra operations, is of low computational complexity. Furthermore, because the error model of tri-axis accelerometers has a similar form to tri-axis magnetometers, this calibration algorithm can also be applied to tri-axis accelerometer calibrations.

## 2. Error Model

To illustrate the calibration algorithm, the error model of a tri-axis magnetometer is established. Similar models have been established in [10,11,12,13,14,15,16]. For convenience, the complete tri-axis error model is discussed again with misalignment errors included. The hysteresis errors, temperature-dependent errors, and time-varying errors will not be discussed in this paper. The output errors are classified into two categories: magnetic field distortions and sensor errors.

### 2.1. Magnetic Field Distortions

The magnetic field distortions are due to the ferromagnetic materials attached to the sensor frame, including the hard iron effect and soft iron effect. The hard iron effect is a constant additional magnetic field produced by permanent magnets on the assembled platform. It is represented as a 3 × 1 vector, denoted as **b***_H_*. The soft iron effect is produced by the magnetization of soft magnets. It exerts effects on the magnitude and orientation of the magnetic field according to the external field orientation, which is represented as a 3 × 3 matrix, denoted as *C_si_*. The *i*-row, *j*-column element in the matrix indicates the influence of the external *j*-direction field to the *i*-direction field.

### 2.2. Sensor Errors

The sensor errors of the tri-axis magnetometer include the zero bias error, scale factor error, non-orthogonal error, misalignment error, and noise. These errors are mainly due to machining and installation defects. The zero bias error makes constant offsets to each axis of the magnetometer, which is represented as a 3 × 1 vector, denoted as **b***_zb_*. The scale factor error comes from the sensitivity inconsistencies of each sensor. It is represented as a 3 × 3 diagonal matrix, denoted as *C_sf_*. The elements on the diagonal represent the sensitivities of each axis sensor. The non-orthogonality between individual sensors introduces an inter-axis coupling output error, which is represented as a 3 × 3 upper triangular matrix, denoted as *C_no_*. In general conditions of small angle errors, the diagonal elements of *C_no_* are close to 1. The misalignment error represents the angular misalignment between the tri-axis magnetometer set and the assembled platform, which is represented as a 3 × 3 unit rotation matrix, denoted as *C_m_*. It can be simplified into an anti-symmetric matrix, with diagonal elements equal to 1. The output noise of individual sensor is assumed as uncorrelated Gaussian white noise. The output noise is represented as a 3 × 1 vector, denoted as **ε**.

Above all, the error model of the tri-axis magnetometer is given as:

(1) $$\begin{array}{cc} m & =C_{no}C_{sf}C_{si}C_{m}(\tilde{m}+b_{H})+b_{zb}+\varepsilon \\ & =K\tilde{m}+b+\varepsilon \\ & =\left[\begin{array}{ccc} k_{11} & k_{12} & k_{13}\\ k_{21} & k_{22} & k_{23}\\ k_{31} & k_{32} & k_{33} \end{array}\right]\left(\left[\begin{array}{c} {\tilde{m}}_{x}\\ {\tilde{m}}_{y}\\ {\tilde{m}}_{z} \end{array}\right]\right)+\left[\begin{array}{c} b_{x}\\ b_{y}\\ b_{z} \end{array}\right]+\left[\begin{array}{c} \varepsilon_{x}\\ \varepsilon_{y}\\ \varepsilon_{z} \end{array}\right] \end{array}$$

In the equation, **m** stands for the magnetometer output vector. *K* stands for the total error coefficient matrix, and **b** stands for the total bias error vector. Note that in this paper, ~ stands for ideal quantities, ^ stands for interpolation quantities, and ⌢ stands for compensated quantities.

## 3. Calibration Algorithm Development

**Figure 1:**
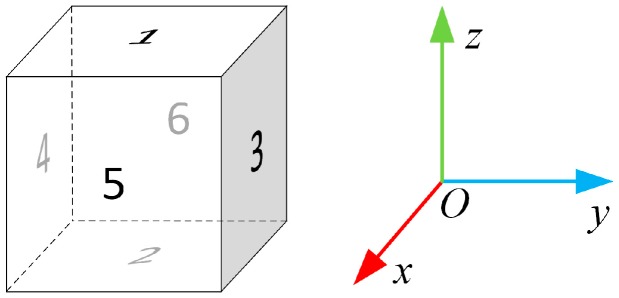
Definition of cuboid sides and sensor set coordinates.

The calibration algorithm is developed for evaluating the error coefficient matrix *K* and the bias error vector **b**. In this calibration algorithm, the sensor set is firstly mounted into a non-magnetic cuboid frame. The six sides of the cuboid are numbered from 1 to 6. Side 1 is opposite to side 2. Side 3 is opposite to side 4, and side 5 is opposite to side 6. The sensor set is aligned with the frame. The *z* axis of the sensor set is perpendicular to side 1 and side 2. The *y* axis of the sensor set is perpendicular to side 3 and side 4. The *x* axis of the sensor set is perpendicular to side 5 and side 6, as shown in Figure 1. The calibration process is divided into two steps. In the first step, the tri-axis magnetometer interpolation outputs at specified spin angular intervals are calculated. In the second step, the error coefficient matrix and bias error vector are calculated through linear algebra operations of magnetometer interpolation outputs.

### 3.1. Magnetometer Interpolation Output Calculation

**Figure 2:**
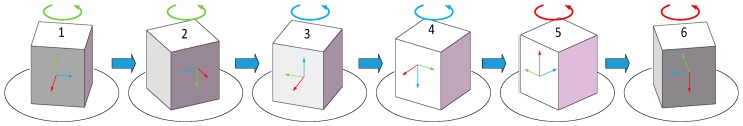
Cuboid frame rotation process.

The cuboid frame is put on a table and rotated over one cycle by hand. This is repeated for each numbered side facing upwards. Then the tri-axis magnetometer output data **m***^k^* and gyroscope output data ***ω****^k^* are obtained, *k* = 1–6, where *k* stands for the number of the upward side. The process is shown in Figure 2. Here are some points to note: (1) the sequence of which side is facing up can be arbitrary; (2) the orientation of the flank when putting the frame on the table can be arbitrary (finding north is not required); (3) both clockwise and anticlockwise rotations are accepted; (4) constant rotation speed is not required; and (5) the number of turns must be larger than 1.

**Figure 3:**
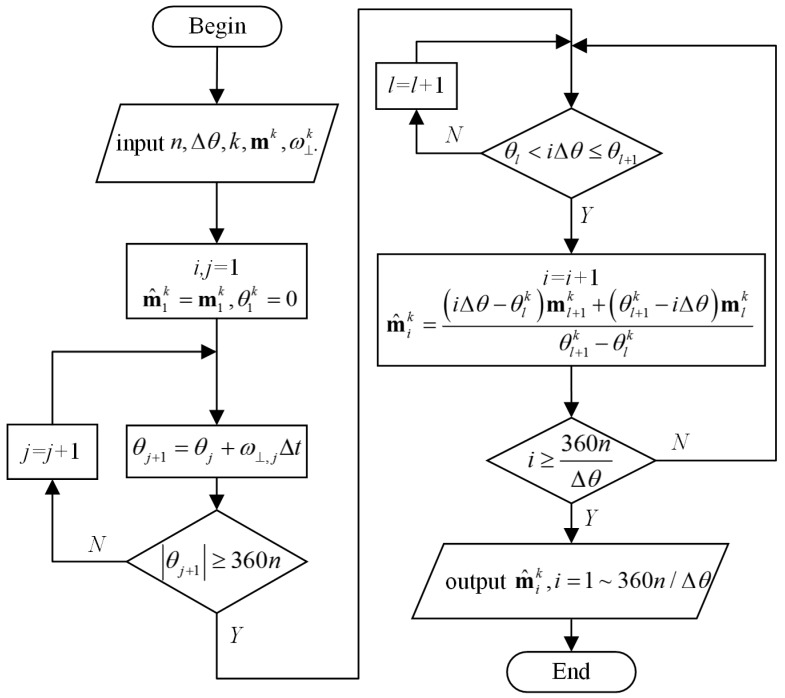
Flowchart for magnetometer output interpolation.

The tri-axis magnetometer interpolation outputs at specified spin angular intervals are calculated from **m***^k^* and ***ω****^k^*. The interpolation flowchart is shown in Figure 3. Firstly, the number of turns *n*, and the angular interval Δ*θ* are set. The value of 360/Δ*θ* must be an integer. The upward side number *k* is to be verified. Next, by integrating the output data $\omega_{⊥}^{k}$ of the gyroscope whose sensitive axis is perpendicular to the table, the rotation angles $\theta^{k}$ of each sample points are derived. After which, the interpolation outputs ${\hat{m}}^{k}$ are derived from $m^{k}$ and $\theta^{k}$. Then, the upward side *k* is changed to a different number, and the program is repeated. Finally, the interpolation outputs ${\hat{m}}_{i}^{k}$ are obtained, where *i* = 1, 2,…, 360*n*/Δ*θ*, *k* = 1, 2,…, 6.

### 3.2. Error Coefficient Calculation

The geomagnetic field is stable. It can hardly change within days and is assumed to be constant in the calibration process. The geomagnetic field component perpendicular to the table is denoted as $h_{⊥}$. When side 1 faces upwards, ${\tilde{m}}_{z}^{1}$ is equal to $h_{⊥}$, and Equation (1) can be rewritten as:

(2) $$\left[\begin{array}{c} {\hat{m}}_{x,i}^{1}\\ {\hat{m}}_{y,i}^{1}\\ {\hat{m}}_{z,i}^{1} \end{array}\right]=K\left[\begin{array}{c} {\tilde{m}}_{x,i}^{1}\\ {\tilde{m}}_{y,i}^{1}\\ h_{⊥} \end{array}\right]+\left[\begin{array}{c} b_{x}\\ b_{y}\\ b_{z} \end{array}\right]=K{\tilde{C}}_{i}^{z}\left[\begin{array}{c} {\tilde{m}}_{x,1}^{1}\\ {\tilde{m}}_{y,1}^{1}\\ h_{⊥} \end{array}\right]+\left[\begin{array}{c} b_{x}\\ b_{y}\\ b_{z} \end{array}\right]$$

where ${\tilde{C}}_{i}^{z}$ is the ideal rotation matrix around *z* axis of sensor set, which is expanded as:

(3) $${\tilde{C}}_{i}^{z}=\left[\begin{array}{ccc} \cos\left(i\Delta \theta \right) & \sin\left(i\Delta \theta \right) & 0\\ -\sin\left(i\Delta \theta \right) & \cos\left(i\Delta \theta \right) & 0\\ 0 & 0 & 1 \end{array}\right]=\left[\begin{array}{c} {\tilde{C}}_{i,1}\\ {\tilde{C}}_{i,2}\\ {\tilde{C}}_{i,3} \end{array}\right]$$

Considering that:

(4) $${\tilde{C}}_{i+180{}^{\circ}/\Delta \theta }^{z}=\left[\begin{array}{ccc} \cos\left(i\Delta \theta +180{}^{\circ}\right) & \sin\left(i\Delta \theta +180{}^{\circ}\right) & 0\\ -\sin\left(i\Delta \theta +180{}^{\circ}\right) & \cos\left(i\Delta \theta +180{}^{\circ}\right) & 0\\ 0 & 0 & 1 \end{array}\right]=\left[\begin{array}{c} -{\tilde{C}}_{i,1}\\ -{\tilde{C}}_{i,2}\\ {\tilde{C}}_{i,3} \end{array}\right]$$

by substituting Equation (4) into Equation (2), there is:

(5) $$\left[\begin{array}{c} {\hat{m}}_{x,i+180{}^{\circ}/\Delta \theta }^{1}\\ {\hat{m}}_{y,i+180{}^{\circ}/\Delta \theta }^{1}\\ {\hat{m}}_{z,i+180{}^{\circ}/\Delta \theta }^{1} \end{array}\right]=KC_{i+180{}^{\circ}/\Delta \theta }^{z}\left[\begin{array}{c} {\tilde{m}}_{x,i}^{1}\\ {\tilde{m}}_{y,i}^{1}\\ h_{⊥} \end{array}\right]+\left[\begin{array}{c} b_{x}\\ b_{y}\\ b_{z} \end{array}\right]=K\left[\begin{array}{c} -{\tilde{m}}_{x,i}^{1}\\ -{\tilde{m}}_{y,i}^{1}\\ h_{⊥} \end{array}\right]+\left[\begin{array}{c} b_{x}\\ b_{y}\\ b_{z} \end{array}\right]$$

Similarly, when side 2 faces upwards, ${\tilde{m}}_{z}^{2}$ is equals to −$h_{⊥}$. There are:

(6) $$\left[\begin{array}{c} {\hat{m}}_{x,i}^{2}\\ {\hat{m}}_{y,i}^{2}\\ {\hat{m}}_{z,i}^{2} \end{array}\right]=K\left[\begin{array}{c} {\tilde{m}}_{x,i}^{2}\\ {\tilde{m}}_{y,i}^{2}\\ -h_{⊥} \end{array}\right]+\left[\begin{array}{c} b_{x}\\ b_{y}\\ b_{z} \end{array}\right]$$

(7) $$\left[\begin{array}{c} {\hat{m}}_{x,i+180{}^{\circ}/\Delta \theta }^{2}\\ {\hat{m}}_{y,i+180{}^{\circ}/\Delta \theta }^{2}\\ {\hat{m}}_{z,i+180{}^{\circ}/\Delta \theta }^{2} \end{array}\right]=K\left[\begin{array}{c} -{\tilde{m}}_{x,i}^{2}\\ -{\tilde{m}}_{x,i}^{2}\\ -h_{⊥} \end{array}\right]+\left[\begin{array}{c} b_{x}\\ b_{y}\\ b_{z} \end{array}\right]$$

When side 3 faces upwards, ${\tilde{m}}_{y}^{3}$ is equals to $h_{⊥}$. There are:

(8) $$\left[\begin{array}{c} {\hat{m}}_{x,i}^{3}\\ {\hat{m}}_{y,i}^{3}\\ {\hat{m}}_{z,i}^{3} \end{array}\right]=K\left[\begin{array}{c} {\tilde{m}}_{x,i}^{3}\\ h_{⊥}\\ {\tilde{m}}_{z,i}^{3} \end{array}\right]+\left[\begin{array}{c} b_{x}\\ b_{y}\\ b_{z} \end{array}\right]$$

(9) $$\left[\begin{array}{c} {\hat{m}}_{x,i+180{}^{\circ}/\Delta \theta }^{3}\\ {\hat{m}}_{y,i+180{}^{\circ}/\Delta \theta }^{3}\\ {\hat{m}}_{z,i+180{}^{\circ}/\Delta \theta }^{3} \end{array}\right]=K\left[\begin{array}{c} -{\tilde{m}}_{x,i}^{3}\\ h_{⊥}\\ -{\tilde{m}}_{z,i}^{3} \end{array}\right]+\left[\begin{array}{c} b_{x}\\ b_{y}\\ b_{z} \end{array}\right]$$

When side 4 faces upwards, ${\tilde{m}}_{y}^{4}$ is equals to −$h_{⊥}$. There are:

(10) $$\left[\begin{array}{c} {\hat{m}}_{x,i}^{4}\\ {\hat{m}}_{y,i}^{4}\\ {\hat{m}}_{z,i}^{4} \end{array}\right]=K\left[\begin{array}{c} {\tilde{m}}_{x,i}^{4}\\ -h_{⊥}\\ {\tilde{m}}_{z,i}^{4} \end{array}\right]+\left[\begin{array}{c} b_{x}\\ b_{y}\\ b_{z} \end{array}\right]$$

(11) $$\left[\begin{array}{c} {\hat{m}}_{x,i+180{}^{\circ}/\Delta \theta }^{4}\\ {\hat{m}}_{y,i+180{}^{\circ}/\Delta \theta }^{4}\\ {\hat{m}}_{z,i+180{}^{\circ}/\Delta \theta }^{4} \end{array}\right]=K\left[\begin{array}{c} -{\tilde{m}}_{x,i}^{4}\\ -h_{⊥}\\ -{\tilde{m}}_{z,i}^{4} \end{array}\right]+\left[\begin{array}{c} b_{x}\\ b_{y}\\ b_{z} \end{array}\right]$$

When side 5 faces upwards, ${\tilde{m}}_{x}^{5}$ is equals to $h_{⊥}$. There are:

(12) $$\left[\begin{array}{c} {\hat{m}}_{x,i}^{5}\\ {\hat{m}}_{y,i}^{5}\\ {\hat{m}}_{z,i}^{5} \end{array}\right]=K\left[\begin{array}{c} h_{⊥}\\ {\tilde{m}}_{y,i}^{5}\\ {\tilde{m}}_{z,i}^{5} \end{array}\right]+\left[\begin{array}{c} b_{x}\\ b_{y}\\ b_{z} \end{array}\right]$$

(13) $$\left[\begin{array}{c} {\hat{m}}_{x,i+180{}^{\circ}/\Delta \theta }^{5}\\ {\hat{m}}_{y,i+180{}^{\circ}/\Delta \theta }^{5}\\ {\hat{m}}_{z,i+180{}^{\circ}/\Delta \theta }^{5} \end{array}\right]=K\left[\begin{array}{c} h_{⊥}\\ -{\tilde{m}}_{y,i}^{5}\\ -{\tilde{m}}_{z,i}^{5} \end{array}\right]+\left[\begin{array}{c} b_{x}\\ b_{y}\\ b_{z} \end{array}\right]$$

When side 6 faces upwards, ${\tilde{m}}_{x}^{6}$ is equals to −$h_{⊥}$. There are:

(14) $$\left[\begin{array}{c} {\hat{m}}_{x,i}^{6}\\ {\hat{m}}_{y,i}^{6}\\ {\hat{m}}_{z,i}^{6} \end{array}\right]=K\left[\begin{array}{c} -h_{⊥}\\ {\tilde{m}}_{y,i}^{6}\\ {\tilde{m}}_{z,i}^{6} \end{array}\right]+\left[\begin{array}{c} b_{x}\\ b_{y}\\ b_{z} \end{array}\right]$$

(15) $$\left[\begin{array}{c} {\hat{m}}_{x,i+180{}^{\circ}/\Delta \theta }^{6}\\ {\hat{m}}_{y,i+180{}^{\circ}/\Delta \theta }^{6}\\ {\hat{m}}_{z,i+180{}^{\circ}/\Delta \theta }^{6} \end{array}\right]=K\left[\begin{array}{c} -h_{⊥}\\ -{\tilde{m}}_{y,i}^{6}\\ -{\tilde{m}}_{z,i}^{6} \end{array}\right]+\left[\begin{array}{c} b_{x}\\ b_{y}\\ b_{z} \end{array}\right]$$

By Equations (2)–(15), the error coefficient matrix *K* and bias error vector **b** are solved through linear operations as follows:

1. By linear operation of Equations (2), (5)–(15), **b** is solved:
  (16) $$\sum_{k=1}^{6}\sum_{i=1}^{360n/\Delta \theta }\left[\begin{array}{c} {\hat{m}}_{x,i}^{k}\\ {\hat{m}}_{y,i}^{k}\\ {\hat{m}}_{z,i}^{k} \end{array}\right]=\frac{2160n}{\Delta \theta }\left[\begin{array}{c} b_{x}\\ b_{y}\\ b_{z} \end{array}\right]$$
2. By linear operation of Equations (2), (5)–(7), $h_{⊥}K_{3}$ is solved:
  (17) $$\sum_{i=1}^{360n/\Delta \theta }\left[\begin{array}{c} {\hat{m}}_{x,i}^{1}\\ {\hat{m}}_{y,i}^{1}\\ {\hat{m}}_{z,i}^{1} \end{array}\right]-\sum_{i=1}^{360n/\Delta \theta }\left[\begin{array}{c} {\hat{m}}_{x,i}^{2}\\ {\hat{m}}_{y,i}^{2}\\ {\hat{m}}_{z,i}^{2} \end{array}\right]=\frac{720n}{\Delta \theta }K\left[\begin{array}{c} 0\\ 0\\ h_{⊥} \end{array}\right]=\frac{720n}{\Delta \theta }h_{⊥}\left[\begin{array}{c} k_{13}\\ k_{23}\\ k_{33} \end{array}\right]$$
3. By linear operation of Equations (8)–(11), $h_{⊥}K_{2}$ is solved:
  (18) $$\sum_{i=1}^{360n/\Delta \theta }\left[\begin{array}{c} {\hat{m}}_{x,i}^{3}\\ {\hat{m}}_{y,i}^{3}\\ {\hat{m}}_{z,i}^{3} \end{array}\right]-\sum_{i=1}^{360n/\Delta \theta }\left[\begin{array}{c} {\hat{m}}_{x,i}^{4}\\ {\hat{m}}_{y,i}^{4}\\ {\hat{m}}_{z,i}^{4} \end{array}\right]=\frac{720n}{\Delta \theta }K\left[\begin{array}{c} 0\\ h_{⊥}\\ 0 \end{array}\right]=\frac{720n}{\Delta \theta }h_{⊥}\left[\begin{array}{c} k_{12}\\ k_{22}\\ k_{32} \end{array}\right]$$
4. By linear operation of Equations (12)–(15), $h_{⊥}K_{1}$ is solved:
  (19) $$\sum_{i=1}^{360n/\Delta \theta }\left[\begin{array}{c} {\hat{m}}_{x,i}^{5}\\ {\hat{m}}_{y,i}^{5}\\ {\hat{m}}_{z,i}^{5} \end{array}\right]-\sum_{i=1}^{360n/\Delta \theta }\left[\begin{array}{c} {\hat{m}}_{x,i}^{6}\\ {\hat{m}}_{y,i}^{6}\\ {\hat{m}}_{z,i}^{6} \end{array}\right]=\frac{720n}{\Delta \theta }K\left[\begin{array}{c} h_{⊥}\\ 0\\ 0 \end{array}\right]=\frac{720n}{\Delta \theta }h_{⊥}\left[\begin{array}{c} k_{11}\\ k_{21}\\ k_{31} \end{array}\right]$$

From Equations (16)–(19), $h_{⊥}K$ and **b** are derived. $h_{⊥}$ is a constant, which makes a scale for the tri-axis outputs. This scale has no effect on the orientation estimations. In many applications, the magnetometer outputs are normalized. Therefore, the tri-axis outputs can be compensated as:

(20) $$\overset{⌢}{m}=\frac{{\left(h_{⊥}K\right)}^{-1}(m-b)}{\left|{\left(h_{⊥}K\right)}^{-1}(m-b)\right|}$$

However, in some applications, such as magnetic field measurements, $h_{⊥}$ needs to be determined. $h_{⊥}$ can be obtained by geomagnetic field models, or by a single-axis calibrated magnetometer. In general cases, the non-diagonal elements of *K* are close to zero, so $h_{⊥}$ can also be evaluated as:

(21) $$h_{⊥}=\frac{\Delta \theta }{2160n}\sum_{i=1}^{360n/\Delta \theta }\left({\hat{m}}_{z,i}^{1}-{\hat{m}}_{z,i}^{2}+{\hat{m}}_{y,i}^{3}-{\hat{m}}_{y,i}^{4}+{\hat{m}}_{x,i}^{5}-{\hat{m}}_{x,i}^{6}\right)$$

Finally, the tri-axis magnetometer outputs are compensated as follows:

(22) $$\overset{⌢}{m}=h_{⊥}{\left(h_{⊥}K\right)}^{-1}(m-b)$$

The program code of the calibration algorithm is presented in the Appendix A.

## 4. Calibration Error Analysis

The gyro errors, cuboid frame errors, and rotation operation errors may degrade the calibration performance. The calibration errors introduced by the gyro bias, gyro misalignment, non-orthogonality of the cuboid frame, and rotation deviation are analyzed, respectively.

### 4.1. Gyro Bias

Gyro bias is a main error of gyroscopes. Most of the bias error can be estimated during stationary time periods, and the residual tri-axis gyroscope bias error is assumed to be:

(23) $$b_{g}={\left[\begin{array}{ccc} b_{gx} & b_{gy} & b_{gz} \end{array}\right]}^{T}$$

When side 1 faces upwards, the magnetometer interpolation outputs are:

(24) $$\left[\begin{array}{c} {\hat{m}}_{x,i}^{1}\\ {\hat{m}}_{y,i}^{1}\\ {\hat{m}}_{z,i}^{1} \end{array}\right]=KC_{i}^{z}\left[\begin{array}{c} {\tilde{m}}_{x,1}^{1}\\ {\tilde{m}}_{y,1}^{1}\\ {\tilde{m}}_{z,1}^{1} \end{array}\right]+\left[\begin{array}{c} b_{x}\\ b_{y}\\ b_{z} \end{array}\right]$$

The actual rotation matrix around the *z* axis is:

(25) $$C_{i}^{z}=\left[\begin{array}{ccc} \cos\left(i\Delta \theta -ib_{gz}\Delta t\right) & \sin\left(i\Delta \theta -ib_{gz}\Delta t\right) & 0\\ -\sin\left(i\Delta \theta -ib_{gz}\Delta t\right) & \cos\left(i\Delta \theta -ib_{gz}\Delta t\right) & 0\\ 0 & 0 & 1 \end{array}\right]$$

Δ*t* is the time interval between two adjacent interpolation points.

The angle measurements by integrating the gyroscope output data are of good accuracy in the short-term, and the angle errors are of small value. The total time during one direction rotation is assumed to be less than 10 s, and by testing the gyroscope in the experiment, the bias error is less than 0.5°/s, which means the angle error estimated by the gyroscope is less than 5°. Under the condition of small angle errors, Equation (25) is rearranged as follows:

(26) $$C_{i}^{z}={\tilde{C}}_{i}^{z}+\Delta C_{i}^{z}$$

where ${\Delta C}_{i}^{z}$ is the rotation matrix error caused by the gyro bias at *i*-th interpolation point, given as:

(27) $$\Delta C_{i}^{z}=\left(ib_{gz}\Delta t\right)\left[\begin{array}{ccc} \sin\left(i\Delta \theta \right) & -\cos\left(i\Delta \theta \right) & 0\\ \cos\left(i\Delta \theta \right) & \sin\left(i\Delta \theta \right) & 0\\ 0 & 0 & 0 \end{array}\right]$$

$\Lambda_{1}$ is defined as the sum of the rotation matrix errors caused by the gyro bias when side 1 faces upwards, derived as:

(28) $$\Lambda_{1}=\sum_{i=1}^{360n/\Delta \theta }\Delta C_{i}^{z}=-\left(\frac{180}{\Delta \theta }b_{gz}n\Delta t\right)\cot\left(\frac{\Delta \theta }{2}\right)\left[\begin{array}{ccc} 1 & 0 & 0\\ 0 & 1 & 0\\ 0 & 0 & 0 \end{array}\right]$$

Similarly, there is:

(29) $$\left\{ \begin{array}{l} \Lambda_{1}=-\left(\frac{180}{\Delta \theta }b_{gz}n\Delta t\right)\cot\left(\frac{\Delta \theta }{2}\right)\left[\begin{array}{ccc} 1 & 0 & 0\\ 0 & 1 & 0\\ 0 & 0 & 0 \end{array}\right],\Lambda_{2}=\left(\frac{180}{\Delta \theta }b_{gz}n\Delta t\right)\cot\left(\frac{\Delta \theta }{2}\right)\left[\begin{array}{ccc} 1 & 0 & 0\\ 0 & 1 & 0\\ 0 & 0 & 0 \end{array}\right]\\ \Lambda_{3}=-\left(\frac{180}{\Delta \theta }b_{gy}n\Delta t\right)\cot\left(\frac{\Delta \theta }{2}\right)\left[\begin{array}{ccc} 1 & 0 & 0\\ 0 & 0 & 0\\ 0 & 0 & 1 \end{array}\right],\Lambda_{4}=\left(\frac{180}{\Delta \theta }b_{gy}n\Delta t\right)\cot\left(\frac{\Delta \theta }{2}\right)\left[\begin{array}{ccc} 1 & 0 & 0\\ 0 & 0 & 0\\ 0 & 0 & 1 \end{array}\right]\\ \Lambda_{5}=-\left(\frac{180}{\Delta \theta }b_{gx}n\Delta t\right)\cot\left(\frac{\Delta \theta }{2}\right)\left[\begin{array}{ccc} 0 & 0 & 0\\ 0 & 1 & 0\\ 0 & 0 & 1 \end{array}\right],\Lambda_{6}=\left(\frac{180}{\Delta \theta }b_{gx}n\Delta t\right)\cot\left(\frac{\Delta \theta }{2}\right)\left[\begin{array}{ccc} 0 & 0 & 0\\ 0 & 1 & 0\\ 0 & 0 & 1 \end{array}\right] \end{array}\right.$$

Thus, the calibration error of **b** caused by gyro bias is derived by applying Equation (29) into Equation (16), as shown in Equation (30):

(30) $$\Delta b_{gb}=\frac{\Delta t}{12}\cot\left(\frac{\Delta \theta }{2}\right)\left(b_{gz}\left[\begin{array}{c} {\tilde{m}}_{x,1}^{1}-{\tilde{m}}_{x,1}^{2}\\ {\tilde{m}}_{y,1}^{1}-{\tilde{m}}_{y,1}^{2}\\ 0 \end{array}\right]+b_{gy}\left[\begin{array}{c} {\tilde{m}}_{x,1}^{3}-{\tilde{m}}_{x,1}^{4}\\ 0\\ {\tilde{m}}_{z,1}^{3}-{\tilde{m}}_{z,1}^{4} \end{array}\right]+b_{gx}\left[\begin{array}{c} 0\\ {\tilde{m}}_{y,1}^{5}-{\tilde{m}}_{y,1}^{6}\\ {\tilde{m}}_{z,1}^{5}-{\tilde{m}}_{z,1}^{6} \end{array}\right]\right)$$

If the initial orientations of the flank when side *k* and side *k* + 1 (*k* = 1, 3, 5) face upwards are the same, the gyro bias error would cause little calibration error to **b**, and if the initial orientation of the flank are opposite, the gyro bias error would cause maximal calibration error to **b**.

The calibration error of *K* caused by the gyro bias is derived by applying Equation (29) into Equations (17)–(19), as shown in Equation (31):

(31) $$\Delta K_{gb}=\frac{\Delta t}{4h_{⊥}}\cot\left(\frac{\Delta \theta }{2}\right)\left[\begin{array}{ccc} 0 & b_{gy}\left({\tilde{m}}_{x,1}^{3}+{\tilde{m}}_{x,1}^{4}\right) & b_{gz}\left({\tilde{m}}_{x,1}^{1}+{\tilde{m}}_{x,1}^{2}\right)\\ b_{gx}\left({\tilde{m}}_{y,1}^{5}+{\tilde{m}}_{y,1}^{6}\right) & 0 & b_{gz}\left({\tilde{m}}_{y,1}^{1}+{\tilde{m}}_{y,1}^{2}\right)\\ b_{gx}\left({\tilde{m}}_{z,1}^{5}+{\tilde{m}}_{z,1}^{6}\right) & b_{gy}\left({\tilde{m}}_{z,1}^{3}+{\tilde{m}}_{z,1}^{4}\right) & 0 \end{array}\right]$$

If the initial orientations of the flank when side *k* and side *k* + 1 (*k* = 1, 3, 5) face upwards are opposite, the gyro bias error would cause little calibration error to *K*, and if the initial orientation of the flank are the same, the gyro bias error would cause maximal calibration error to *K*.

${\Delta b}_{\mathrm{gb}}$ and ${\Delta K}_{\mathrm{gb}}$ satisfy:

(32) $$\left\{ \begin{array}{l} \left|\Delta b_{gb}\right|\leq \frac{\Delta t}{6}\cot\left(\frac{\Delta \theta }{2}\right)b_{g}F\cos\left(I\right){\left[\begin{array}{ccc} 1 & 1 & 1 \end{array}\right]}^{T}\\ \left|\Delta K_{gb}\right|\leq \frac{\Delta t}{2}\cot\left(I\right)\cot\left(\frac{\Delta \theta }{2}\right)b_{g}\left[\begin{array}{ccc} 0 & 1 & 1\\ 1 & 0 & 1\\ 1 & 1 & 0 \end{array}\right] \end{array}\right.$$

where *F* is the total geomagnetic field intensity, and *I* is the geomagnetic inclination angle.

Considering *I* = 59°, Δ*t* = 0.02 s, Δ*θ* = 1°, $b_{g}$ = 0.5°/s, and that the magnetometer outputs are normalized, even under the worst initial orientation conditions, the elements of ${\Delta b}_{\mathrm{gb}}$ and ${\Delta K}_{\mathrm{gb}}$ are less than 0.0034 and 0.0060, respectively.

### 4.2. Gyro Misalignment

When installing the gyroscope into the cuboid frame, the misalignment between gyro axes and cuboid faces could be introduced. This would affect the gyro outputs, and cause estimation errors of the rotation angle. The gyro misalignment is presented as a rotation matrix of the small angle:

(33) $$C_{gm}=\left[\begin{array}{ccc} 1+o\left(1\right) & -\psi & \theta \\ \psi & 1+o\left(1\right) & -\phi \\ -\theta & \phi & 1+o\left(1\right) \end{array}\right]$$

*o*(∙) denotes the infinitesimal. The gyro output errors are:

(34) $$\left[\begin{array}{c} \Delta g_{1}\\ \Delta g_{2}\\ \Delta g_{⊥} \end{array}\right]=\left(C_{gm}-I_{3}\right)\left[\begin{array}{c} 0\\ 0\\ \omega \end{array}\right]=\left[\begin{array}{c} \theta \omega \\ -\phi \omega \\ o\left(\omega \right) \end{array}\right]$$

${\Delta g}_{1}$, ${\Delta g}_{2}$ are the gyro output errors whose axes are parallel to the table, and ${\Delta g}_{⊥}$ is the gyro output error whose axis is perpendicular to the table. The misalignment angle causes little error on $g_{⊥}$, which is applied to rotation estimation.

Thus, the gyro misalignment error introduces slight calibration errors, which can be ignored.

### 4.3. Non-Orthogonality of Cuboid Frame

**Figure 4:**
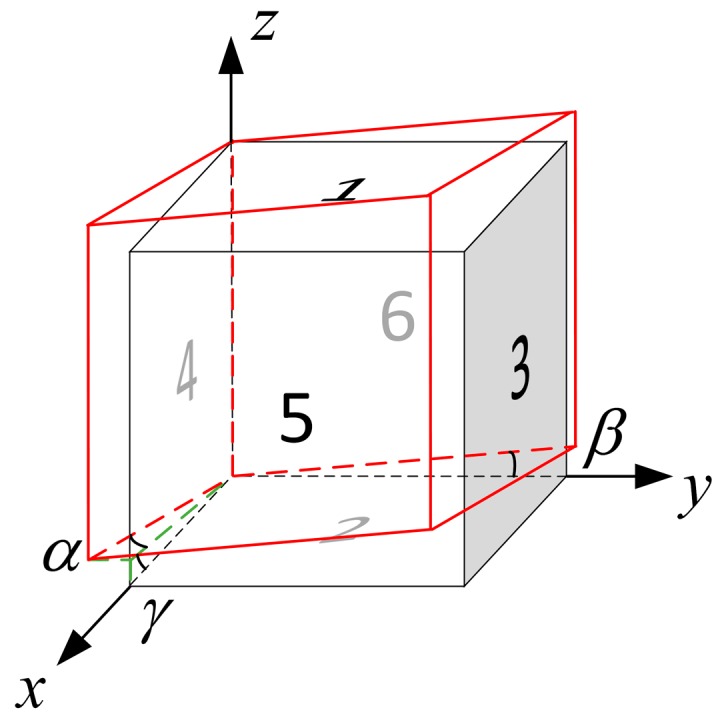
Non-orthogonality of the cuboid frame.

Non-orthogonality of the cuboid frame is indicated in Figure 4. The *α*, *β* and *γ* are the deviation angles, which are of small value.

When sides 1–6 are perpendicular to the table, respectively, the output errors of the gyroscope whose axis is perpendicular to the table are derived, respectively:

(35) $$\left\{ \begin{array}{l} \Delta g_{no,⊥}^{1,2}=\omega_{⊥}^{1,2}\left(\cos\beta -1\right)=o\left(\beta \right)\\ \Delta g_{no,⊥}^{3,4}=\omega_{⊥}^{3,4}\left(\cos\alpha -1\right)=o\left(\alpha \right)\\ \Delta g_{no,⊥}^{5,6}=\omega_{⊥}^{5,6}\left(\cos\alpha \cos\gamma -1\right)=o\left(\alpha \right)+o\left(\gamma \right) \end{array}\right.$$

It can be deduced from Equation (35) that the non-orthogonality of the cuboid frame has little effect on the vertical gyro outputs.

The magnetometer output error, caused by the non-orthogonality of the cuboid frame, is represented when side 1 faces upwards:

(36) $$\Delta m_{no,i}^{1}=\left(\left[\begin{array}{ccc} 1 & 0 & 0\\ 0 & 1 & \beta \\ 0 & -\beta & 1 \end{array}\right]\left[\begin{array}{ccc} 1 & 0 & -\gamma \\ 0 & 1 & 0\\ \gamma & 0 & 1 \end{array}\right]-I_{3}\right)\left[\begin{array}{c} {\tilde{m}}_{x,i}^{1}\\ {\tilde{m}}_{y,i}^{1}\\ h_{⊥} \end{array}\right]=\left[\begin{array}{c} -\gamma h_{⊥}\\ \beta h_{⊥}\\ \gamma {\tilde{m}}_{x,i}^{1}-\beta {\tilde{m}}_{y,i}^{1} \end{array}\right]$$

In the same way, the magnetometer output errors with each side upwards are derived as:

(37) $$\left\{ \begin{array}{l} \Delta m_{no,i}^{1}={\left[\begin{array}{ccc} -\gamma h_{⊥} & \beta h_{⊥} & \gamma {\tilde{m}}_{x,i}^{1}-\beta {\tilde{m}}_{y,i}^{1} \end{array}\right]}^{T},\Delta m_{no,i}^{2}={\left[\begin{array}{ccc} \gamma h_{⊥} & -\beta h_{⊥} & \gamma {\tilde{m}}_{x,i}^{2}-\beta {\tilde{m}}_{y,i}^{2} \end{array}\right]}^{T}\\ \Delta m_{no,i}^{3}={\left[\begin{array}{ccc} \alpha h_{⊥} & -\alpha {\tilde{m}}_{x,i}^{3} & 0 \end{array}\right]}^{T},\Delta m_{no,i}^{4}={\left[\begin{array}{ccc} -\alpha h_{⊥} & -\alpha {\tilde{m}}_{x,i}^{4} & 0 \end{array}\right]}^{T}\\ \Delta m_{no,i}^{5}={\left[\begin{array}{ccc} -\gamma {\tilde{m}}_{z,i}^{5} & 0 & \gamma h_{⊥} \end{array}\right]}^{T},\Delta m_{no,i}^{6}={\left[\begin{array}{ccc} -\gamma {\tilde{m}}_{z,i}^{6} & 0 & -\gamma h_{⊥} \end{array}\right]}^{T} \end{array}\right.$$

Thus, by applying Equations (37) into Equation (16), the calibration error of **b** caused by the non-orthogonality of the cuboid frame is calculated:

(38) $$\Delta b_{no}=\frac{\Delta \theta }{2160n}\sum_{i=1}^{360n/\Delta \theta }\left(\Delta m_{no,i}^{1}+\Delta m_{no,i}^{2}+\Delta m_{no,i}^{3}+\Delta m_{no,i}^{4}+\Delta m_{no,i}^{5}+\Delta m_{no,i}^{6}\right)={\left[\begin{array}{ccc} 0 & 0 & 0 \end{array}\right]}^{T}$$

By applying Equation (37) into Equations (17)–(19), the calibration error of *K* is calculated:

(39) $$\Delta K_{no}=\frac{\Delta \theta }{720nh_{⊥}}\sum_{i=1}^{360n/\Delta \theta }\left[\begin{array}{ccc} \Delta m_{no,i}^{5}-\Delta m_{no,i}^{6} & \Delta m_{no,i}^{3}+\Delta m_{no,i}^{4} & \Delta m_{no,i}^{1}-\Delta m_{no,i}^{2} \end{array}\right]=\left[\begin{array}{ccc} 0 & \alpha & -\gamma \\ 0 & 0 & \beta \\ \gamma & 0 & 0 \end{array}\right]$$

The non-orthogonality error of the cuboid frame introduces calibration errors to four elements of *K*, and does not affect the other elements of *K* and **b**. The errors are equal to the deviation angles. Assuming the machining verticality is 0.02, which is easy to be satisfied, and the side length of the cuboid frame is 100 mm, the errors to the four elements of *K* are less than 2 × 10^−4^, which could be ignored.

### 4.4. Rotation Deviation

While rotating the frame, the orientation of the rotations may change slightly due to the table being uneven and vibrations caused by friction during the rotation. This can result in unwanted effects on the gyro outputs and magnetometer outputs. The orientation deviation is presented as:

(40) $$C_{rd}=\left[\begin{array}{ccc} 1+o\left(1\right) & 0 & -\varepsilon_{1}\\ 0 & 1 & 0\\ \varepsilon_{1} & 0 & 1+o\left(1\right) \end{array}\right]\left[\begin{array}{ccc} 1 & 0 & 0\\ 0 & 1+o\left(1\right) & \varepsilon_{2}\\ 0 & -\varepsilon_{2} & 1+o\left(1\right) \end{array}\right]=\left[\begin{array}{ccc} 1+o\left(1\right) & o\left(1\right) & -\varepsilon_{1}\\ 0 & 1+o\left(1\right) & \varepsilon_{2}\\ \varepsilon_{1} & -\varepsilon_{2} & 1+o\left(1\right) \end{array}\right]$$

The gyro output error caused by the rotation deviation is:

(41) $$\left[\begin{array}{c} \Delta g_{rd,1}\\ \Delta g_{rd,2}\\ \Delta g_{rd,⊥} \end{array}\right]=\left(C_{rd}-I_{3}\right)\left[\begin{array}{c} 0\\ 0\\ \omega \end{array}\right]+\left[\begin{array}{c} o\left(\omega \right)\\ o\left(\omega \right)\\ 0 \end{array}\right]=\left[\begin{array}{c} o\left(\omega \right)-\varepsilon_{1}\omega \\ o\left(\omega \right)+\varepsilon_{2}\omega \\ o\left(\omega \right) \end{array}\right]$$

$g_{⊥}$ is slightly affected by the rotation deviation.

When side 1 faces upwards, the magnetometer output errors caused by the rotation deviation is:

(42) $$\Delta m_{rd,i}^{1}=\left(C_{rd,i}-I_{3}\right)\left[\begin{array}{c} {\tilde{m}}_{x,i}^{1}\\ {\tilde{m}}_{y,i}^{1}\\ h_{⊥} \end{array}\right]=\left[\begin{array}{c} -\varepsilon_{1,i}^{1}h_{⊥}\\ \varepsilon_{2,i}^{1}h_{⊥}\\ \varepsilon_{1,i}^{1}{\tilde{m}}_{x,i}^{1}-\varepsilon_{2,i}^{1}{\tilde{m}}_{y,i}^{1} \end{array}\right]$$

In the same way, the magnetometer output errors when the other sides face upwards are obtained:

(43) $$\left\{ \begin{array}{l} \Delta m_{rd,i}^{1}={\left[\begin{array}{ccc} -\varepsilon_{1,i}^{1}h_{⊥} & \varepsilon_{2,i}^{1}h_{⊥} & \varepsilon_{1,i}^{1}{\tilde{m}}_{x,i}^{1}-\varepsilon_{2,i}^{1}{\tilde{m}}_{y,i}^{1} \end{array}\right]}^{T},\Delta m_{rd,i}^{2}={\left[\begin{array}{ccc} \varepsilon_{1,i}^{2}h_{⊥} & -\varepsilon_{2,i}^{2}h_{⊥} & \varepsilon_{1,i}^{2}{\tilde{m}}_{x,i}^{2}-\varepsilon_{2,i}^{2}{\tilde{m}}_{y,i}^{2} \end{array}\right]}^{T}\\ \Delta m_{rd,i}^{3}={\left[\begin{array}{ccc} -\varepsilon_{2,i}^{3}h_{⊥} & \varepsilon_{1,i}^{3}{\tilde{m}}_{x,i}^{3}-\varepsilon_{2,i}^{3}{\tilde{m}}_{z,i}^{3} & \varepsilon_{1,i}^{3}h_{⊥} \end{array}\right]}^{T},\Delta m_{rd,i}^{4}={\left[\begin{array}{ccc} \varepsilon_{2,i}^{4}h_{⊥} & \varepsilon_{1,i}^{4}{\tilde{m}}_{x,i}^{4}-\varepsilon_{2,i}^{4}{\tilde{m}}_{z,i}^{4} & -\varepsilon_{1,i}^{4}h_{⊥} \end{array}\right]}^{T}\\ \Delta m_{rd,i}^{5}={\left[\begin{array}{ccc} \varepsilon_{1,i}^{5}{\tilde{m}}_{x,i}^{5}-\varepsilon_{2,i}^{5}{\tilde{m}}_{y,i}^{5} & \varepsilon_{1,i}^{5}h_{⊥} & -\varepsilon_{2,i}^{5}h_{⊥} \end{array}\right]}^{T},\Delta m_{rd,i}^{6}={\left[\begin{array}{ccc} \varepsilon_{1,i}^{6}{\tilde{m}}_{x,i}^{6}-\varepsilon_{2,i}^{6}{\tilde{m}}_{y,i}^{6} & -\varepsilon_{1,i}^{6}h_{⊥} & \varepsilon_{2,i}^{6}h_{⊥} \end{array}\right]}^{T} \end{array}\right.$$

By applying Equation (43) into Equation (16), the calibration error of **b** caused by the rotation deviation is calculated:

(44) $$\Delta b_{rd}=\frac{\Delta \theta }{2160n}\sum_{i=1}^{360n/\Delta \theta }\left(\Delta m_{rd,i}^{1}+\Delta m_{rd,i}^{2}+\Delta m_{rd,i}^{3}+\Delta m_{rd,i}^{4}+\Delta m_{rd,i}^{5}+\Delta m_{rd,i}^{6}\right)$$

By applying Equation (43) into Equations (17)–(19), the calibration error of *K* is calculated:

(45) $$\Delta K_{rd}=\frac{\Delta \theta }{720nh_{⊥}}\sum_{i=1}^{360n/\Delta \theta }\left[\begin{array}{ccc} \Delta m_{rd,i}^{5}-\Delta m_{rd,i}^{6} & \Delta m_{rd,i}^{3}+\Delta m_{rd,i}^{4} & \Delta m_{rd,i}^{1}-\Delta m_{rd,i}^{2} \end{array}\right]$$

The deviation angles $\varepsilon_{j,i}^{k}$ are assumed to be normally distributed with zero mean and variance of $Q_{\varepsilon }$, and the $\varepsilon_{j,i}^{k}$ and $m_{j,i}^{k}$ are uncorrelated. The variance of the calibration error caused by the rotation deviation satisfies:

(46) $$\left\{ \begin{array}{l} Var\left(\Delta b_{rd}\right)<\frac{F^{2}\Delta \theta Q}{3240n}{\left[\begin{array}{ccc} 1 & 1 & 1 \end{array}\right]}^{T}\\ Var\left(\Delta K_{rd}\right)<\frac{\Delta \theta Q}{720n}\left[\begin{array}{ccc} 2\cot^{2}\left(I\right) & 1 & 1\\ 1 & 2\cot^{2}\left(I\right) & 1\\ 1 & 1 & 2\cot^{2}\left(I\right) \end{array}\right] \end{array}\right.$$

The magnetometer outputs are considered as being normalized, such that the norm of the magnetic field *F* is set as 1. If $\sqrt{Q}$ = 1°, *I* = 59°, Δ*θ* = 1°, and *n* = 2, the standard deviations of the elements in ${\Delta b}_{\mathrm{rd}}$ are less than 1.2 × 10^−3^, and the standard deviations of diagonal elements and off-diagonal elements in ${\Delta K}_{\mathrm{rd}}$ are less than 2.2 × 10^−3^ and 2.6 × 10^−3^, respectively.

Above all, the calibration errors introduced by the gyro bias, gyro misalignment, non-orthogonality of the cuboid frame, and rotation deviation are slight and acceptable under normal conditions.

## 5. Simulation

To illustrate the calibration accuracy and computational requirements of the proposed method, the ellipsoid-fitting calibration method in [13] is performed for comparison. Hereafter, the ellipsoid-fitting calibration method in [13] is referred to as the EM, to distinguish from the proposed calibration method is referred to as the PM.

The local magnetic field is acquired from the world magnetic model [26]. As an example, the north, east, and down components of the Beijing area are 27,959.6 nT, −3296.3 nT, and 46,697.7 nT, respectively. Considering the sensor set used in the following experiment, the magnetometer output noise is set to 200 nT (1σ). The gyroscope output noise is 0.05°/s (1σ) and the zero drift is 0.5°/s for each individual sensor. The sample frequency is 100 Hz. The number of turns *n* is set to 2, and the interpolation angular interval Δ*θ* is set to 1°.

Two error conditions of the tri-axis magnetometer are considered for further comparison. In the error condition 1, all of the tri-axis magnetometer errors are included. In the error condition 2, the misalignment error, soft iron error, and non-orthogonality error of the magnetometer, which cause rotations of the output data and degrade the calibration performance of the EM, are removed.

### 5.1. Error Condition 1

The magnetometer errors are set and shown in Table 1.

Then the ideal total error coefficient matrix and total bias error vector are derived:

$$\tilde{K}=\left[\begin{array}{ccc} 1.0780 & -0.1465 & -0.1529\\ 0.0845 & 0.9149 & -0.1107\\ 0.2112 & 0.1655 & 1.1250 \end{array}\right],\;\tilde{b}=\left[\begin{array}{c} 7133.44\\ 1668.75\\ -976.57 \end{array}\right]$$

By applying the PM, the total error coefficient matrix *K*_PM_ and total bias error vector **b**_PM_ are calculated, as shown below. The average element estimation errors of *K*_PM_ and **b**_PM_ are 0.0008 and 24 nT, respectively.

$$K_{PM}=\left[\begin{array}{ccc} 1.0778 & -0.1460 & -0.1511\\ 0.0838 & 0.9148 & -0.1121\\ 0.2134 & 0.1651 & 1.1251 \end{array}\right],\;b_{PM}=\left[\begin{array}{c} 7108.58\\ 1657.84\\ -1013.90 \end{array}\right]$$

By applying the EM, the total error coefficient matrix *K*_EM_ and total bias error vector **b**_EM_ are calculated as below. The average element estimation errors of *K*_2_ and **b**_2_ are 0.1010, and 94 nT, respectively.

$$K_{EM}=\left[\begin{array}{ccc} 1.0903 & -0.0066 & 0.0071\\ -0.0066 & 0.9175 & 0.0108\\ 0.0071 & 0.0108 & 1.1473 \end{array}\right],\;b_{EM}=\left[\begin{array}{c} 7167.78\\ 1770.43\\ -1121.99 \end{array}\right]$$

**Figure 5:**
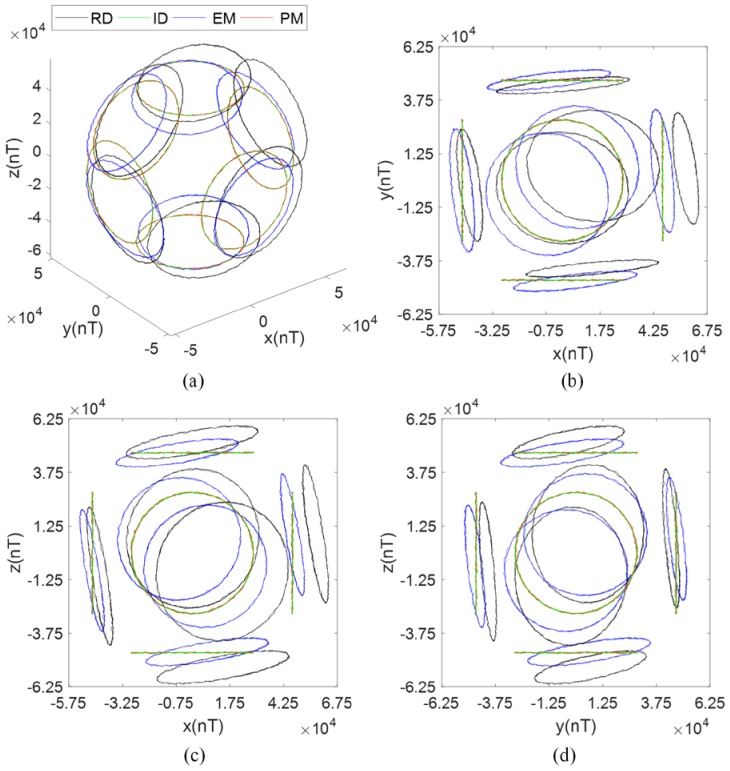
Magnetometer output data (simulation under condition 1): (**a**) Isometric view; (**b**) *z*-axis view; (**c**) *y*-axis view; (**d**) *x*-axis view.

**Figure 6:**
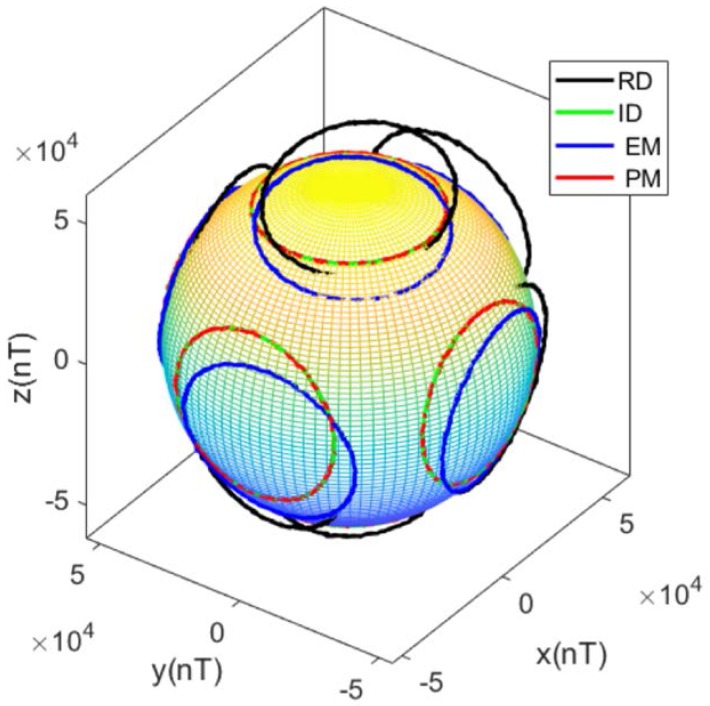
Magnetometer output data and fitting sphere (simulation under condition 1).

The tri-axis magnetometer output data during the calibration process are plotted in Figure 5, where RD refers to raw data, and ID refers to ideal data. After calibration using the PM, the calibrated outputs are consistent with the ideal outputs. The distorted ellipses are rectified into circles, centered at [0, 0], and parallel to the sides of the cuboid, which means the hard iron effect, soft iron effect, zero bias error, scale factor error, non-orthogonal error, and misalignment error are compensated. After calibration using the EM, as shown in Figure 6, the distorted ellipses are rectified into circles, and the calibrated outputs are distributed on the surface of a sphere centered at [0, 0, 0], which means that the total scale error and total zero bias error, introduced by the scale factor error, zero bias error, soft iron error, and non-orthogonality error, are compensated. However, the calibrated outputs have a rotation error from the ideal outputs. The rotation error has the same matrix form as the misalignment error, which is considered as a total misalignment error introduced by the misalignment error, soft iron error, and non-orthogonality error [12].

**Figure 7:**
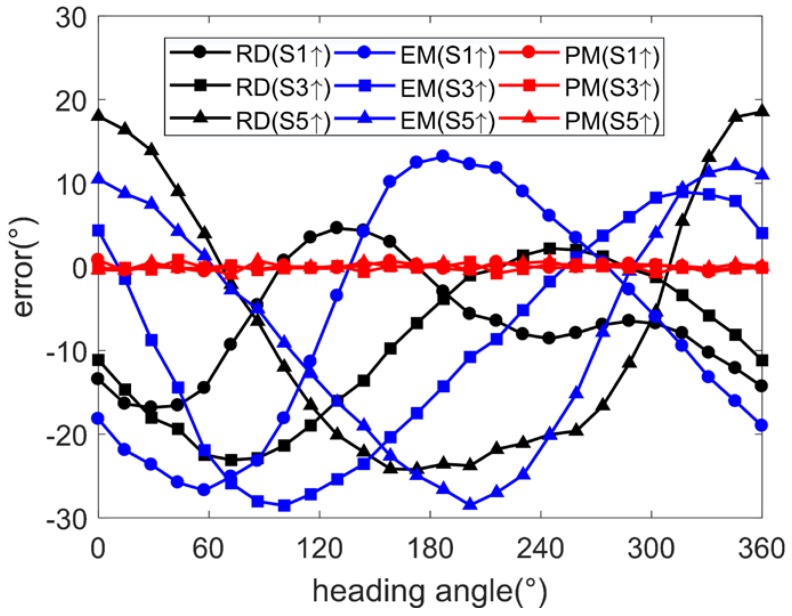
Angle error (simulation under condition 1).

Using the arctangent function, the heading angles are calculated from the output data of two-axis magnetometers parallel to the table. When side 1 faces upwards, the heading angle is calculated by the *x*- and *y*-axis magnetometer outputs. When side 3 faces upwards, the heading angle is calculated by the *x*- and *z*-axis magnetometer outputs. When side 5 faces upwards, the heading angle is calculated by the *y*- and *z*-axis magnetometer outputs. By comparing the raw and calibrated angle errors under the conditions of side 1, side 3, and side 5 facing upwards, respectively, the calibration performances of the EM and PM are illustrated, as shown in Figure 7.

After calibration using the EM, the angle errors are not reduced, but such errors become more consistent when different sides face upwards. After calibration using the PM, the angle errors are limited to 0.5° (1σ), with the maximum less than 1.5°, as shown in Table 2.

### 5.2. Error Condition 2

**Figure 8:**
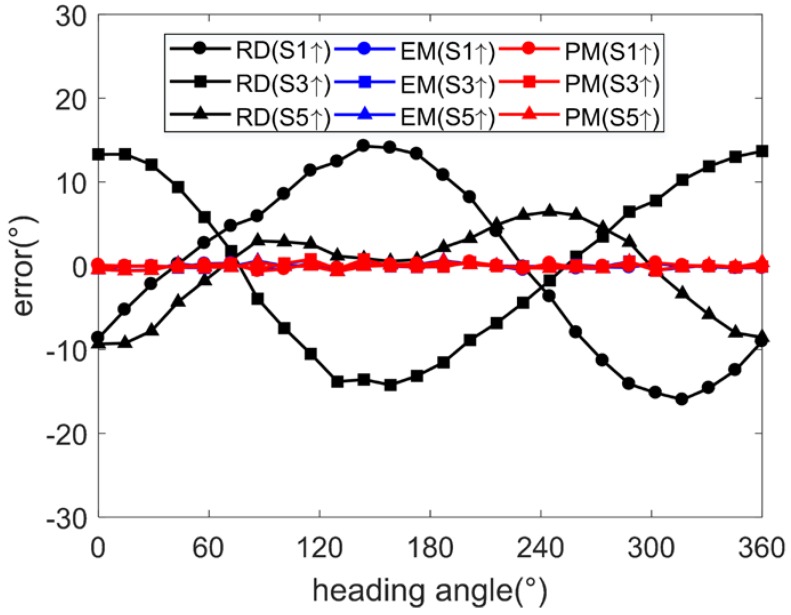
Angle errors (simulation under condition 2).

Under error condition 2, the misalignment error *C_m_*, soft iron error *C_si_*, and non-orthogonality error *C_no_* of the magnetometer are removed and the other parameters keep the same. The simulation program is repeated under this condition. The angle errors are eliminated effectively both using the EM and the PM, as shown in Figure 8 and Table 3. When there is no misalignment error, soft iron error, and non-orthogonality error, this shows that the EM performs efficiently. The PM performs efficiently under both error conditions.

The amounts of computation of the EM and PM are compared. Both the EM and PM are divided into two steps. In the EM, step 1 is to perform ellipsoid-fitting using least squares technique, and step 2 is to extract the error coefficients from the ellipsoid parameters. In the PM, step 1 is to calculate the magnetometer interpolation outputs using the linear interpolation method, and step 2 is to calculate the error coefficients from the interpolation outputs by linear operations. The number of data for ellipsoid fitting is set to be equal to the number of interpolation outputs, which is equal to 360*n*/Δ*θ*. The simulation program is repeated ten times under both of the error conditions using Matlab, and the average computation time of these two algorithms is shown in Table 4. The step 1 in the EM requires large amounts of computation. The linear operations in the PM requires small computational amounts. The EM requires computations amounting to more than 10 times that of the PM.

In summary, three conclusions are drawn: (1) using the proposed calibration method, all of the magnetometer errors are compensated, including the hard iron effect, soft iron effect, zero bias error, scale factor error, non-orthogonal error, and misalignment error; (2) using the standalone ellipsoid-fitting calibration algorithm, the total scale factor error and the total zero bias error are compensated, but the total misalignment error is uncompensated; and (3) the computational requirements of the proposed method is much lower than the computational requirements of the ellipsoid-fitting method.

## 6. Experiment

In this experiment, an SBG IG-500N MARG unit is used. As specified in the manual, the norm of the tri-axis magnetometer output vector is approximately equal to 1 with an arbitrary unit. The noise characteristics of the magnetometer and gyroscope are tested first. The magnetometer output noise is about 0.006 (1σ). The gyroscope output noise and bias are about 0.07°/s (1σ) and 0.5°/s, respectively, and the gyro bias errors are not compensated in advance. The sample frequency is set to be 100 Hz.

**Figure 9:**
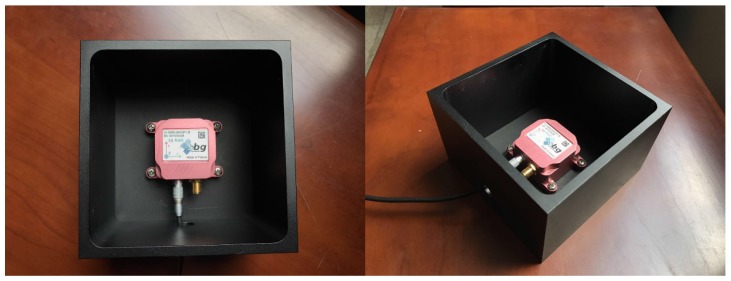
Cuboid frame with the mounted sensor set.

The unit is mounted to a cuboid frame as shown in Figure 9. The number of turns *n* is set to 2, and the interpolation angular interval Δ*θ* is set to 1°. The frame is put on a flat wooden table, and rotated by hand over *n* cycles for each side of the frame.

By applying the PM, the total error coefficient matrix and total bias error vector are calculated as follows:

$$K_{PM}=\left[\begin{array}{ccc} 1.0757 & -0.0343 & 0.0214\\ -0.0426 & 0.9534 & 0.0426\\ 0.0199 & 0.0747 & 0.9709 \end{array}\right],b_{PM}=\left[\begin{array}{c} -0.0427\\ 0.1020\\ 0.0314 \end{array}\right]$$

By applying the EM, the total error coefficient matrix and total bias error vector are calculated as follows:

$$K_{EM}=\left[\begin{array}{ccc} 1.0923 & -0.0203 & 0.0110\\ -0.0203 & 0.9630 & 0.0299\\ 0.0110 & 0.0299 & 0.9863 \end{array}\right],b_{EM}=\left[\begin{array}{c} -0.0396\\ 0.0973\\ 0.0253 \end{array}\right]$$

**Figure 10:**
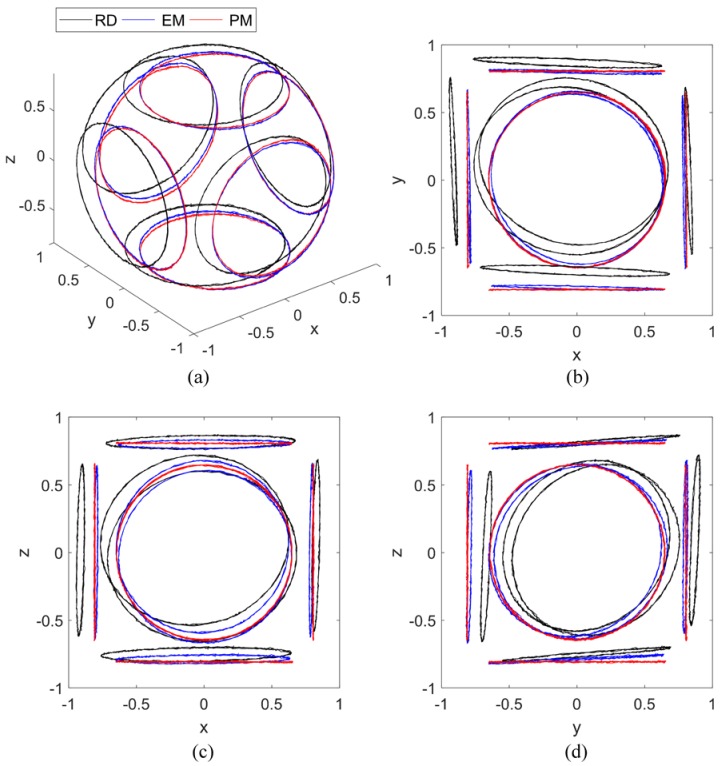
Magnetometer output data (experiment): (**a**) Isometric view; (**b**) *z*-axis view; (**c**) *y*-axis view; (**d**) *x*-axis view.

The raw and calibrated tri-axis magnetometer output data during the calibration process are plotted in Figure 10. After calibration using the PM, the data lie on the circles parallel to the sides of the cuboid, centered at (0, 0). After calibration using the EM, the data lie on the circles unparalleled to the sides of cuboid, which means that the misalignment error still exists. The results of the experiment are consistent with the results of the simulation.

**Figure 11:**
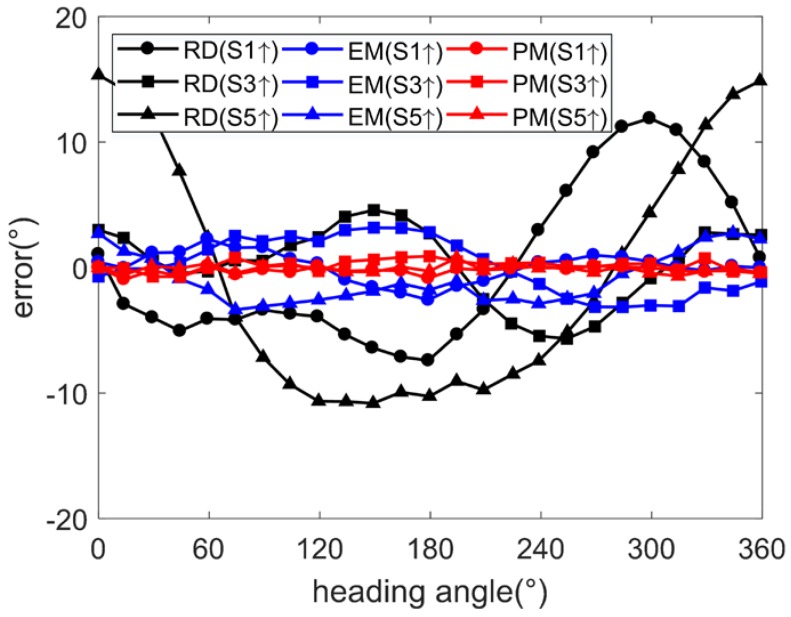
Angle errors (experiment).

For further verification of the quality of the calibrations, the frame is installed on a turntable. By choosing the installation location, the magnetic disturbance from the turntable is reduced to a low level. The turntable is rotated over 1 cycle. Assuming the output angle from turntable as the ideal heading angle, the angle errors after calibration using the EM and PM are derived under the conditions of side 1 upwards, side 3 upwards and side 5 upwards, respectively, as shown in Figure 11.

As shown in Table 5, by applying the EM, the angle errors are narrowed to 2.5° (1σ) with the maximum less than 4.2°. By applying the PM, the angle errors are narrowed to 0.5° (1σ) with the maximum less than 1.7°.

The average computation time is shown in Table 6. The EM requires computations amounting to more than 10 times that of the PM, which is consistent with the simulation results.

## 7. Conclusions

This article proposed a complete tri-axis magnetometer calibration algorithm with a gyroscope auxiliary. A non-magnetic cuboid frame was required to mount the sensor set, and was rotated by hand during the calibration, which was inexpensive and easy to operate.

The calibration errors introduced by the gyro bias error, gyro misalignment error, cuboid frame non-orthogonality error, and rotation deviation error were analyzed, respectively. The results of the analyses indicated that the calibration errors were slight and acceptable.

The computation time of the proposed method was compared with the ellipsoid-fitting calibration method, which indicated that the linear operations provided this algorithm with low computational complexity.

The calibration results of the ellipsoid-fitting method and the proposed method were compared using simulations and experiments. The tri-axis magnetometer output errors were calibrated using the proposed method, including the hard iron effect, soft iron effect, zero bias error, scale factor error, non-orthogonal error, and misalignment error, while the total misalignment error, introduced by the misalignment error, soft iron error, and non-orthogonality error, was unable to be calibrated under the stand-alone ellipsoid fitting calibration algorithms. The results from the simulations and experiments were consistent. After calibration using the proposed method, the heading error was reduced to 0.5° (1σ).

## Figures and Tables

**Table 1:** Tri-axis magnetometer errors.

Error	Value	Error	Value
$C_{m}$	$\left[\begin{array}{ccc} 1 & -0.01 & 0.03\\ 0.01 & 1 & 0.02\\ -0.03 & 0.02 & 1 \end{array}\right]$	$C_{no}$	$\left[\begin{array}{ccc} 1 & -0.04 & 0.02\\ 0 & 1 & 0.01\\ 0 & 0 & 1 \end{array}\right]$
$C_{sf}$	$\left[\begin{array}{ccc} 1.08 & 0 & 0\\ 0 & 0.95 & 0\\ 0 & 0 & 1.10 \end{array}\right]$	$C_{si}$	$\left[\begin{array}{ccc} 0.99 & -0.10 & -0.20\\ 0.07 & 0.96 & -0.15\\ 0.22 & 0.13 & 1.02 \end{array}\right]$
$b_{H}$	$\left[\begin{array}{ccc} 6000 & 2000 & -3000 \end{array}\right]\left(nT\right)$	$b_{zb}$	$\left[\begin{array}{ccc} 500 & -1000 & 800 \end{array}\right]\left(nT\right)$

**Table 2:** Angle error (simulation under condition 1).

Calibration Method	Side 1 Upwards (°)			Side 3 Upwards (°)			Side 5 Upwards (°)		
	E	σ	max	E	σ	max	E	σ	max
**Raw**	−6.61	6.32	17.60	−9.38	8.71	23.44	−7.70	15.20	24.95
**EM**	−6.46	13.84	27.00	−9.31	13.04	28.80	−7.76	13.84	28.38
**PM**	−0.01	0.44	1.36	−0.03	0.37	1.13	0.05	0.40	1.29

**Table 3:** Angle error (simulation under condition 2).

Calibration Method	Side 1 Upwards (°)			Side 3 Upwards (°)			Side 5 Upwards (°)		
	E	σ	max	E	σ	max	E	σ	max
**Raw**	−0.03	10.08	16.18	−0.03	9.92	14.90	−0.03	4.71	9.44
**EM**	−0.02	0.41	1.37	−0.03	0.38	1.08	−0.03	0.37	1.19
**PM**	−0.03	0.41	1.34	−0.03	0.38	1.07	−0.01	0.39	1.36

**Table 4:** Computation time (simulation, CPU: Intel i7 @3.6 GHz, RAM: 8 GB).

Calibration Method	Step 1	Step 2	Total
**EM**	0.3549s	0.0012s	**0.3561s**
**PM**	0.0161s	0.0014s	**0.0175s**

**Table 5:** Angle errors (experiment).

Calibration Method	Side 1 Upwards (°)			Side 3 Upwards (°)			Side 5 Upwards (°)		
	E	σ	max	E	σ	max	E	σ	max
**Raw**	−2.14	5.00	12.03	1.42	2.83	6.17	3.82	11.04	16.01
**EM**	0.18	0.97	2.58	0.53	2.12	4.14	0.26	2.32	3.68
**PM**	−0.11	0.45	1.63	0.32	0.49	1.66	−0.10	0.34	1.17

**Table 6:** Computation time (experiment, CPU: Intel i7 @3.6 GHz, RAM: 8 GB).

Calibration Method	Step 1	Step 2	Total
**EM**	0.2177s	0.0011s	**0.2188s**
**PM**	0.0163s	0.0011s	**0.0174s**

## Appendix A

The code of the proposed method is written for Matlab. Related data are posted online (https://www.researchgate.net/profile/Deng_Yang3/publication/316968794_Data_of_magnetometer_ang_gyro_for_Complete_Tri-Axis_Magnetometer_Calibration_with_Gyro_Auxiliary/data/591ae41aaca272fa34dbac01/MGData-YD.mat)

**Algorithm** Complete Tri-Axis Magnetometer Calibration with a Gyro Auxiliary
function [K,b,Mint]=MagCal_YD(MGdata,n,dA,dt,ht)
% K: total error coefficient matrix
% b: total bias error vector
% Mint: tri-axis magnetometer interpolation outputs
% MGdata: j x l x k matrix of tri-axis gyro and magnetometer outputs
% j is the j-th sample point
% l=1~6, represent the x-, y-, z-axis gyro outputs with unit of degree/s, and x-, y-, z-axis magnetometer outputs, respectively.
% k=1~6, represent the sensor direction of z-axis upward, z-axis downward, y-axis upward, y-axis downward, x-axis upward, x-axis downward, respectively.
% n: number of rotations, n>=1.
% dA: angular interval with unit of degree. 360/dA must be an integer.
% dt: sampling period, with unit of s.
% ht: the geomagnetic field component perpendicular to the table. If ht is unknow, set ht=0.
%% step 1: Magnetometer Interpolation Output Calculation
AR=360*n;
ni= AR/dA;
Mint=zeros(AR/dA,3,6);
num=length(MGdata(:,1,1));
angle=zeros(3*num,6);
for k=1:6
i=1;j=1;l=1;
while abs(angle(j,k))<AR
angle(j+1,k)=angle(j,k)+MGdata(j,4-floor(0.5*k+0.5),k)*dt;
j=j+1;
end
if angle(j,k)>0
angle(1:j,k)=angle(1:j,k);
else
angle(1:j,k)=-angle(1:j,k);
end
Mint(1,:,k)=MGdata(1,4:6,k);
while i<(AR/dA)
if angle(l,k)<(i)*dA && angle(l+1,k)>=(i)*dA
Mint(i+1,:,k)=(i*dA-angle(l,k))/(angle(l+1,k)-angle(l,k))*MGdata(l+1,4:6,k)+(angle(l+1,k)-i*dA)/(angle(l+1,k)-angle(l,k))*MGdata(l,4:6,k);
i=i+1;
else
l=l+1;
end
end
end
%% step 2: Error Coefficient Calculation
if ht==0
ht=1*(sum(Mint(:,3,1))-sum(Mint(:,3,2))+sum(Mint(:,2,3))-sum(Mint(:,2,4))+sum(Mint(:,1,5))-sum(Mint(:,1,6)))/(ni*6);
end
bx=sum(sum(Mint(:,1,:)))/(ni*6);
by=sum(sum(Mint(:,2,:)))/(ni*6);
b=sum(sum(Mint(:,3,:)))/(ni*6);
b=[bx;by;bz];
K=zeros(3,3);
K(:,3)=(sum(Mint(:,1:3,1))-sum(Mint(:,1:3,2)))'/(ni*2*ht);
K(:,2)=(sum(Mint(:,1:3,3))-sum(Mint(:,1:3,4)))'/(ni*2*ht);
K(:,1)=(sum(Mint(:,1:3,5))-sum(Mint(:,1:3,6)))'/(ni*2*ht);
end
